# Berbamine, a bioactive alkaloid, suppresses equine herpesvirus type 1 *in vitro* and *in vivo*

**DOI:** 10.3389/fvets.2023.1163780

**Published:** 2023-05-25

**Authors:** Zeyu Li, Yuanxiu He, Lijuan Ge, Ran Quan, Junzhen Chen, Yue Hu, Ruixue Sa, Jianhua Liu, Duoliang Ran, Qiang Fu, Huijun Shi

**Affiliations:** College of Veterinary Medicine, Xinjiang Agricultural University, Urumqi, Xinjiang, China

**Keywords:** berbamine, equine herpesvirus-1 (EHV-1), Syrian hamsters, virus replication, tissue injury

## Abstract

Equine herpesvirus type 1 (EHV-1) poses a global threat to equines. The anticancer agent berbamine (BBM), a bioactive alkaloid, has been shown to inhibit viral infection. However, whether BBM can inhibit EHV-1 infection remains unclear. This study investigated the effect of BBM treatment on EHV-1 infection. Quantitative PCR (qPCR), immunoblotting, the Reed-Muench method, and pathological examination were employed to study the ability of BBM to inhibit EHV-1 infection, viral DNA replication, viral protein production, virion secretion, and cytopathogenesis *in vitro* and *in vivo*. The *in vitro* studies revealed that 10 μM BBM effectively suppressed EHV-1 viral entry into cells, viral DNA replication, and virion secretion, while the *in vivo* studies verified the ability of BBM to suppress EHV-1-induced damage of brain and lung tissues and animal mortality. These findings strongly suggest that BBM could be a serious contender in the therapeutic control of EHV-1 infection of equines.

## 1. Introduction

Equine herpesvirus type 1 (EHV-1) is a pathogen that must be reported to the World Organization for Animal Health (OIE) to ensure the safe international trade of horses (1). It is a severe and ubiquitous equine viral pathogen that can cause several diseases/conditions, including respiratory infection (also called rhinopneumonitis) associated with pyrexia, cough, and respiratory distress; miscarriage and stillbirth in pregnant mares; neonatal death; and neurological disorders, with symptoms ranging from mild ataxia to complete paralysis. EHV-1-induced neurological disease is usually referred to as equine herpesvirus myeloencephalopathy (EHM) (2). Although EHM is a relatively uncommon manifestation of EHV-1 infection, it can cause devastating losses and severely impact the equine industry (3). The development of a vaccine against these equine viruses, involving classical and modern vaccinology approaches, has been ongoing for more than five decades, but progress is slow compared with the development of vaccines for other alphaherpesviruses of veterinary importance, such as those affecting cattle and pigs. EHV-1 is still persistent among domesticated horses around the world (4), and currently available vaccines are not completely protective, especially against EHM, and have no specific effect on EHV-1-related conditions (5). Furthermore, although a vaccine is being developed in the US and Europe (6–8), there is no effective drug to treat EHV-1 infection. Drugs of anti-disease were eager to be a new way to reduce and preventive virus inflection.

Berbamine (BBM) is a traditional Chinese herbal medicine extracted from the roots, root bark, and stem bark of *Berberis amurensis* (9). BBM can enhance hematopoietic function and increase the quantity of blood cells (10). It is used to control tumors and leukopenia caused by radiotherapy and chemotherapy, benzene poisoning, and radioactive substances (11, 12). Increasing evidence indicates that BBM has antiviral activities that suppress the infection of positive-strand ribonucleic acid (RNA) viruses, including enterovirus A71 (EV-A71), Japanese encephalitis virus (JEV), Zika virus (ZIKV), Dengue virus (DENV), and Middle East respiratory syndrome coronavirus (MERS-CoV) (13, 14). However, it is unclear whether BBM is effective in controlling EHV-1 infection.

Several animal models for EHV-1 infection have been developed, with many of them using mice and Syrian hamsters (15). Suckling hamsters were primarily used for virus isolation purposes and to study the potential effects of the equine abortion virus, later known as EHV-1 (16). Subsequently, EHV-1 pathogenesis has been evaluated in hamsters. Based on the antiviral effect of BBM, we hypothesized that this compound would confer resistance to the effects of EHV-1 *in vitro* and in the Syrian hamster. This study combined *in vitro* and Syrian hamster models to investigate the effects of BBM on the inhibition of EHV-1 infection, which may provide a new theoretical basis for the prevention and treatment of EHV-1.

## 2. Materials and methods

### 2.1. Standard curve for detecting EHV-1

The EHV-1 specific primer pair referred to the EHV-1 sequence. The DNA standards constructed had a size of 302 bases (nt. 1,207 to 1,509 EHV-1 sequences). The amplified product was purified and cloned into the pMD19-T vector (Takara Biotechnology, Japan) for sequencing. Conversion to genome equivalents was done using the following equations: optical density at 260 nm (OD_260_) of 1 = 50 μg/mL and 1 bp = 660 g/mol. This resulted in a molecular mass for the plasmids (2,994 bp) of 9 × 10^12^ g/mol. For the standard curve, a 10-fold dilution series of the plasmid pEHV-1 was included in each test; based on the temperature of 57°C, the plasmid was diluted to 10^4^, 10^5^,10^6^, 10^7^, 10^8^, 10^9^, and 10^10^ copies/μL, which has an excellent linear relationship with cycle threshold (CT) values. All experiments were conducted using three replicates with the templates, and the fluorescence intensity of the corresponding CT value was used to obtain the amplification curve (17).

### 2.2. Cytotoxicity analysis

BBM was dissolved in dimethyl sulfoxide (DMSO; Sigma, United States) and diluted to 100 mM. Next, 96-well plates containing 5 × 10^4^ RK-13 cells cultured in Dulbecco’s modified eagle medium (DMEM; Biological Industries, Israel) with 10% fetal bovine serum (FBS; Biological Industries) were treated with different doses of BBM (1, 5, 8, 10, 15, and 20 μM) (18) for 8 h at 37°C; 0 μM BBM was used as a negative control. Subsequently, 3–2,5-diphenyl tetrazolium bromide solution (MTT; Sigma) at 5 mg/mL was added to each well and the cells were incubated for another 4 h at 37°C. The culture medium was then aspirated, the wells were washed with phosphate-buffered saline (PBS) and allowed to dry, and 200 μL DMSO was added to each well. The OD was read with a multi-well microplate reader at 570 nm (19). Cell viability was expressed as a percentage of the negative control group. For this part, we operated in the shadows to avoid light falling on the experiment. The experiments were performed in triplicate and expressed as mean ± SD (*p < 0.05).

### 2.3. Determination of proliferating virus titer

In 6-well plates (1 × 10^5^ RK-13 cells/well), cell suspension was collected which treated with DMSO or 10 μM of BBM for 8 h at 37°C, infected with 0.7 MOI of EHV-1, and observed at 0, 12, 24, 36, and 48 h using an inverted microscope. Virus underwent freezing and thawing three times and was reinfected in 96-well plates (5 × 10^4^ RK-13 cells/well) using the Reed–Muench method to calculate the 50% tissue culture infective dose (TCID_50_) for 7 d (20).

### 2.4. Quantitative PCR (qPCR)

The specific primer pair for qPCR was designed using Oligo 6.0 and the EHV-1 glycoprotein B (gB) gene sequence (GenBank accession number: M36298). The primer pair (forward, 5′-GCCATACGTCCCTGTCCGACAA-3; reverse, 5′-CCTCCACCTCCTCGACGATGC-3′) yielded a product of 302 bases (nt. 1,207 to 1,509 EHV-1 sequences) under PCR cycle conditions of 95°C for 2 min, 95°C for 5 s, 60°C for 30 s, 95°C for 15 s, 60°C for 1 min, 95°C for 30 s, and 60°C for 15 s. RK-13 cells were seeded in 6-well plates (1 × 10^5^ cells/well), treated with 10 μM BBM for 8 h at 37°C, and infected with a neuropathogenic strain of EHV-1 (YM2019 kindly provided by Prof. Duoliang Ran (21), GenBank: MT063054.1) at an MOI of 0.7. After 4 h, the media was changed for fresh DMEM. At 0, 12, 24, 36, and 48 h later, suspension DNA was extracted for cells provided with the E.Z.N.A^®^ Viral DNA Kit (D3892-01, Omega Bio-Tek, the United States), and quantified to 6.07 × 10^5^ copies/mL using a QuantiNova SYBR Green PCR Kit (No. 208054) and 7,500 Fast Real-Time PCR software (22).

### 2.5. Western blotting

Western blotting was performed, as previously described in 3.3, on RK-13 cells treated with BBM and infected with EHV-1 at an MOI of 0.7. Harvested RK-13 cells were lysed with RIPA lysis buffer (containing 1 mM PMSF). The protein samples were separated by 10% sodium dodecyl sulfate-polyacrylamide gel electrophoresis (SDS-PAGE) and transferred to polyvinylidene fluoride (PVDF) membranes. The membranes were blocked in 5% skim milk for 2 h and then incubated with primary antibodies against EHV-1 (1:1,000 EHV-1 positive serum) (23) and GAPDH (1,1,000; No. 10494-1-AP, Proteintech Group, United States) at 4°C overnight, followed by incubation with secondary antibodies at room temperature. Blots were exposed using an enhanced chemiluminescence detection kit (P0018S, Beyotime, China) and a chemiluminescence immunoassay analyzer. Relative blot intensity (ratio to GAPDH) was quantified using ImageJ software.

### 2.6. Binding and internalization assay

Binding was studied by adding EHV-1 at an MOI of 0.7 to chilled RK-13 cells. After 0, 2, 4, 6, and 8 h of incubation at 4°C, the cells were washed extensively with chilled PBS, and then lysed. For internalization, the cells were washed extensively with chilled PBS following incubation with EHV-1 for 3 h, and the temperature was changed to 37°C to allow internalization of the attached virus. At 3, 4, 5, and 6 h post-internalization, the cells were washed extensively with acidic PBS to remove cell surface-attached viruses. Total DNA of each lysate sample was collected with the E.Z.N.A^®^ Viral DNA Kit (24).

### 2.7. Attenuation of EHV-1 infection

To evaluate the role of BBM in EHV-1 infection of Syrian hamsters, 48 specific-pathogen-free (SPF) male Syrian hamsters (age 3 weeks, purchased from Charles River, Beijing, China) were randomly divided into three concentration groups and one no-infection group (received 0 mg/kg BBM only). The BBM concentrations were configured as 0 mg/kg (DMSO 660 μL, PEG300 3,960 μL, Tween80 1,320 μL, H_2_O 7,260 μL), 50 mg/kg (BBM 66 mg, DMSO 660 μL, PEG300 3,960 μL, Tween80 1,320 μL, H_2_O 7,260 μL), and 100 mg/kg, (BBM 132 mg, DMSO 660 μL, PEG300 3,960 μL, Tween80 1,320 μL, H_2_O 7,260 μL). The hamsters were given the indicated concentration of BBM by oral administration every 24 h for 3, 7, and 14 d, and infection with 0.1 mL DMEM containing10^9^ TCID_50_ of EHV-1 by nasal drip (23); 0 mg/kg BBM with and without EHV-1 infection were the positive and negative controls, respectively. Brain, lung, intestine, spleen, liver, and kidney tissue samples (25) were collected and 0.2 g of each tissue was ground and cracked, then the DNA was extracted, quantified to 6.07 × 10^5^ copies/mL, analyzed detected using qPCR. Animals were cared for according to the principles outlined in the National Institutes of Health Guide for the Care and Use of Laboratory Animals.

### 2.8. Reduction of prior EHV-1 infection

To detect the effects of BBM on existing EHV-1 infection, 48 Syrian hamsters were randomly divided into two BBM concentration groups (0 or 100 mg/kg) and one no-infection group (received 0 mg/kg BBM only, as negative control). Hamsters were infected with 0.1 mL DMEM containing 10^9^ TCID_50_ of EHV-1 by nasal drip 3 d. After 48 h, BBM was orally administered to each hamster every 24 h for 0 (positive control) 3, 7, and 14 d. Brain, lung, liver, kidney, intestine, and spleen samples were collected. Brain and lung histopathologic changes were detected by microscope. DNA was quantified in the collected tissue samples to measure attenuation of EHV-1 infection. All procedures were approved by the Animal Welfare and Ethics Committee of Xinjiang Agricultural University.

### 2.9. Histology

Tissues were fixed in 10% neutral buffered formalin for 48 h and processed using routine methods for histology. Next, 5-mm sections were stained with hematoxylin and eosin (H&E). Central nervous system sections were taken from the olfactory bulb (OB); piriform cortex; septostriatal, rostral, and caudal diencephalon; rostral mesencephalon; and cerebellum with cerebellar peduncles. Histological lesions were classified as mild, moderate, or severe (26). The severity of the histopathological changes in EHV-1 infected hamsters was investigated. All evaluations were performed by investigators blinded to the treatment and were conducted separately by two pathologists. Histopathological severity was scored such that higher numbers reflected a higher degree of tissue inflammation, injury, and necrosis. Severity was determined by the extent of necrotic cells and the number and relative area of inflammatory cell infiltration.

### 2.10. Statistical analysis

All statistical analyses were conducted using SPSS 17.0. A *t*-test was used to evaluate distribution type. Normally distributed data are expressed as mean ± standard deviation (SD), and an independent sample *t*-test was used for comparisons between the two cohorts.

## 3. Results

### 3.1. Establishment of a standard curve for detecting EHV-1

**Figure 1:**
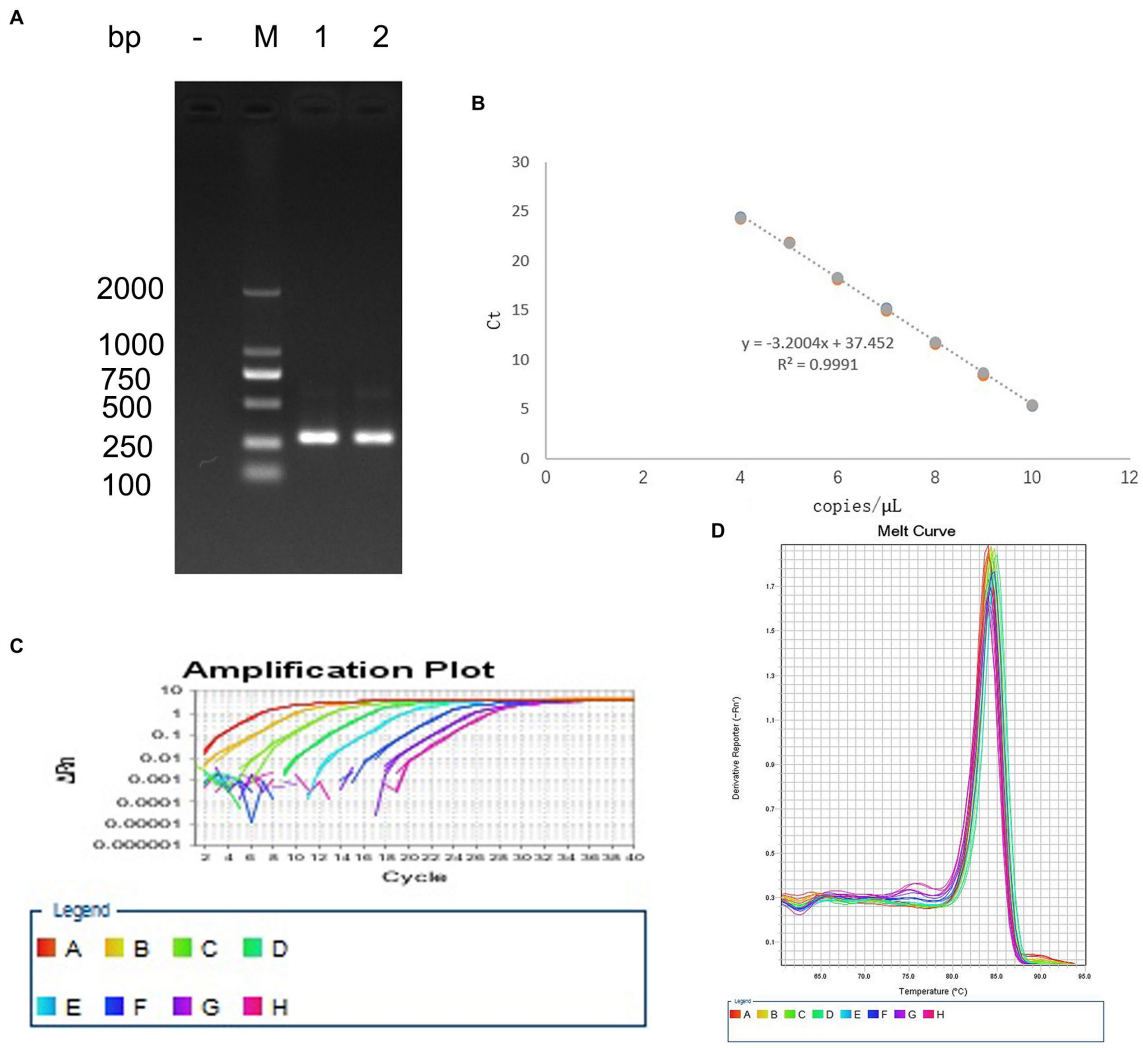
Establishment of the standard curve for detecting EHV-1. **(A)** PCR amplification of the positive plasmid. **(B)** Standard curve. **(C)** Melting curve. **(D)** Amplification curve.

The bands in agarose gel electrophoresis following PCR amplification of EHV-1 DNA extracts indicated that the PCR product was 302 bp (Figure 1A), which was consistent with the expected product size. The coincidence rate of the sequence of positive plasmids was 99%, similar to the sequences documented in the GenBank database. The regression equation of the standard curve was Y = −3.0523X + 36.138, Y(CT), X(Copies), *R*^2^ = 0.9991 (Figure 1B). Amplification efficiency was 113.066%. The melting curve was a single peak (Figure 1C), and the amplification curve was obtained using T_m_ values between 84.5 and 86 (Figure 1D).

### 3.2. BBM inhibited EHV-1 replication *in vitro*

**Figure 2:**
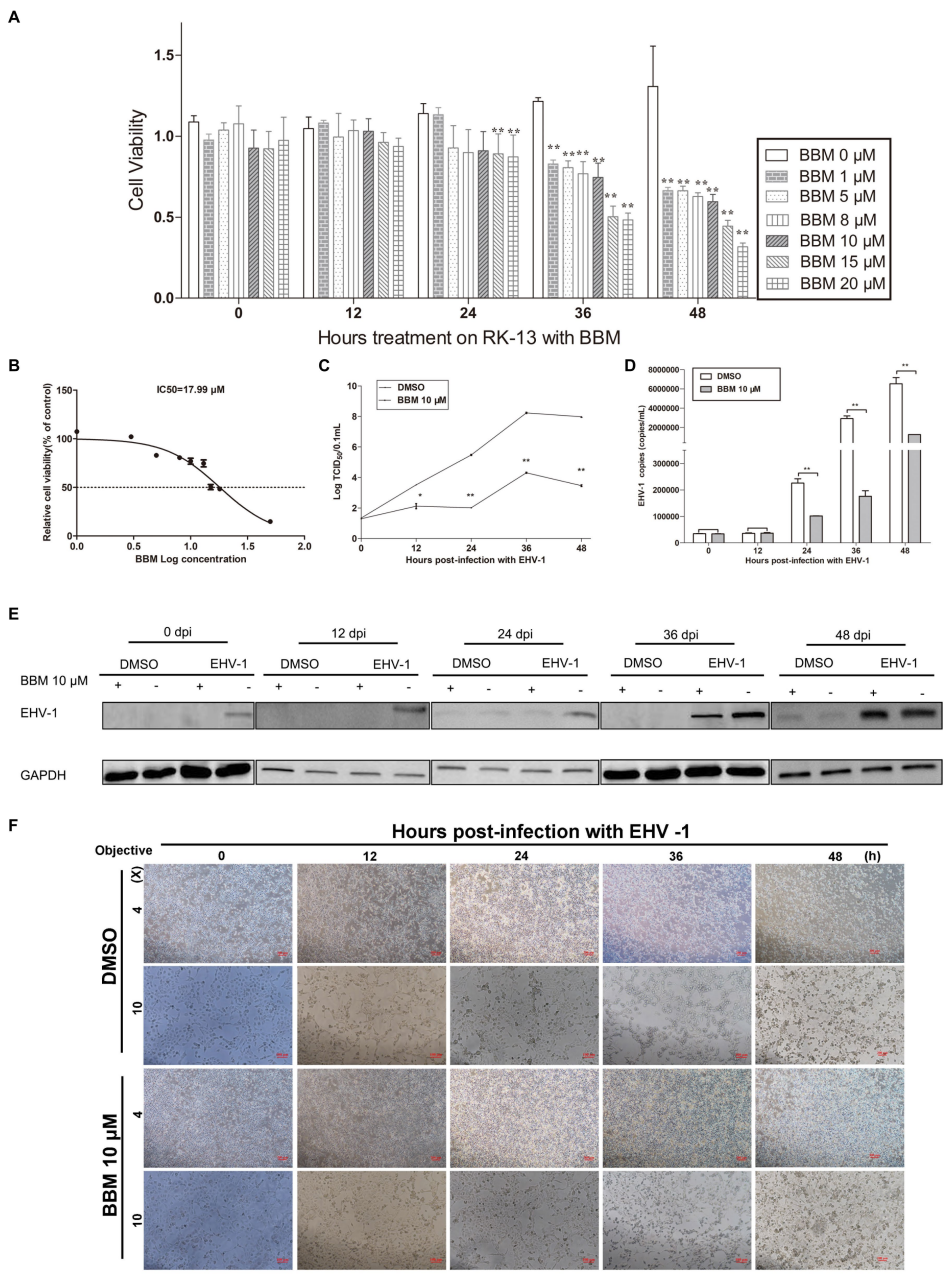
BBM inhibited EHV-1 replication *in vitro*. **(A)** Effect of BBM on the viability of RK-13 cells. **(B)** IC_50_ of BBM on the viability of RK-13 cells. **(C)** Virus titer at different time intervals after EHV-1 infection. **(D)** Detection of EHV-1 DNA accumulation using qPCR at 12, 24, 36, and 48 h pi. **(E)** Western blotting was used to detect the expression of EHV-1 in RK-13 cells at 0, 12, 24, 36, and 48 h pi. **(F)** Detection of the cytopathogenic effect of RK-13 cells. *P*-values less than 0.05 were considered as statistically significant. *p < 0.05 means significant difference, **p < 0.01 means very significant.

An MTT assay was used to reveal the effects of BBM on the viability of RK-13 cells. There was a significant reduction in the viability of the RK-13 cells following treatment with 15 μM and 20 μM BBM for 24 h (*p* < 0.01) (Figure 2A). BBM decreased the viability of the RK-13 cells in a concentration-dependent manner; the IC_50_ of BBM was 17.99 μM (Figure 2B).

To explore the effect of BBM on EHV-1 DNA replication, the anti-EHV-1 activity of BBM was investigated in RK-13 cells. BBM significantly inhibited viral generation from 24 h to 48 h post-infection (pi) (*p* < 0.01) in virus titration assays (Figure 2C) and as quantified in qPCR assays (Figure 2D). From 24 h to 48 h, viral generation decreased about 50 times compared with DMSO treatment.

To determine whether BBM could inhibit increased levels of EHV-1 protein in cells infected with EHV-1, RK-13 cells were treated with 10 μM BBM and infected with EHV-1 for 0, 12, 24, 36, and 48 h. Western blot results showed that BBM significantly inhibited EHV-1 compared with DMSO treatment (Figure 2E).

Next, the cytopathic effect (CPE) of EHV-1 infection was evaluated in the RK-13 cells that were treated with BBM compared with those that were not treated. A CPE was exhibited from 12 h pi in the cells that were not treated with BBM, while BBM treatment reduced cell death and syncytia formation (Figure 2F), and delayed cell death until at least 24 h pi. These data suggest that EHV-1 infection results in the development of a syncytial plaque-forming phenotype in RK-13 cells, but BBM could delay this change, particularly at 24, 36, and 48 h pi.

### 3.3. BBM inhibited EHV-1 infection by repressing viral internalization and binding

**Figure 3:**
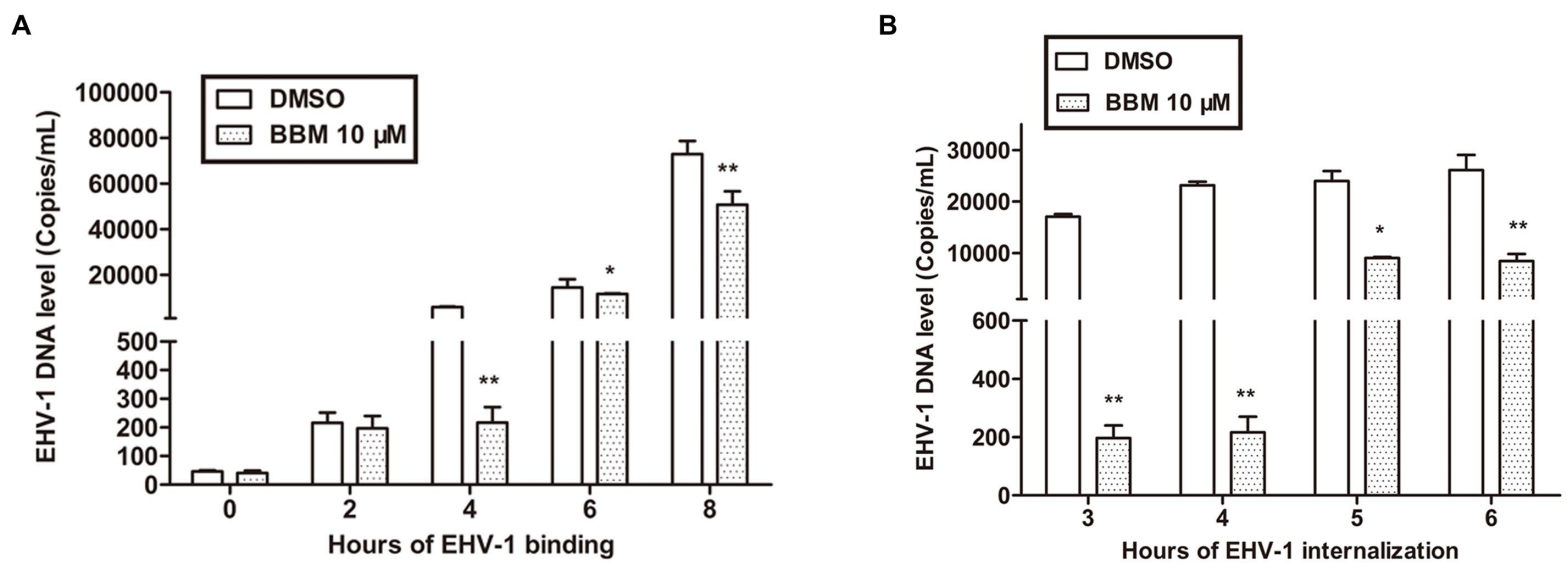
BBM inhibited EHV-1 infection by repressing viral binding and internalization. **(A,B)** Detection of EHV-1 banding and internalization using qPCR. *P*-values less than 0.05 were considered as statistically significant. *p < 0.05 means significant difference, **p < 0.01 means very significant.

To illustrate the mechanism of BBM inhibition of EHV-1, a binding assay was used. RK-13 cells were infected with EHV-1 at 4°C for different durations. Attached viral DNA copies were determined by qPCR, and percentage changes in DNA copies were derived by comparing the number of DNA copies in the BBM samples with that of the untreated cells. In parallel, EHV-1 was pre-blocked or controlled with 10 μM BBM for 1 h at 37°C before inoculating onto chilled RK-13. BBM inhibited EHV-1 infection by repressing viral binding. This effect was related to time, and the qPCR results demonstrated that the number of bound EHV-1 DNA copies was significantly reduced by BBM at 4 h and 8 h pi (*p* < 0.01) compared with controls, and was also reduced at 6 h (*p* < 0.05). As shown in Figure 3A, there was no significant difference in bound EHV-1 DNA copies between BBM and the controls at 0 h and 2 h (*p* > 0.05); however, at 4 h pi, there was an approximate 100-fold decrease in EHV-1 DNA copies in BBM-treated cells compared with control. This indicated that BBM repressed viral binding.

Viral internalization was also explored to explain BBM inhibition of EHV-1 replication. For the internalization assays, we pre-treated RK-13 cell with BBM and used different incubation times. The number of internalized viral DNA copies was determined by qPCR. BBM inhibited EHV-1 infection by repressing viral internalization at all times examined in the assay (3, 4, 5, and 6 h). Figure 3B shows that, compared with controls, the number of internalized EHV-1 DNA copies treated with BBM significantly decreased at different intervals pi (*p* < 0.01), particularly at 4 h incubation where a decrease of approximately 100 times was detected. This indicated that BBM had antiviral activity that inhibited viral internalization from EHV-1 infection.

### 3.4. Attenuating effects of BBM in EHV-1 infection *in vivo*

**Figure 4:**
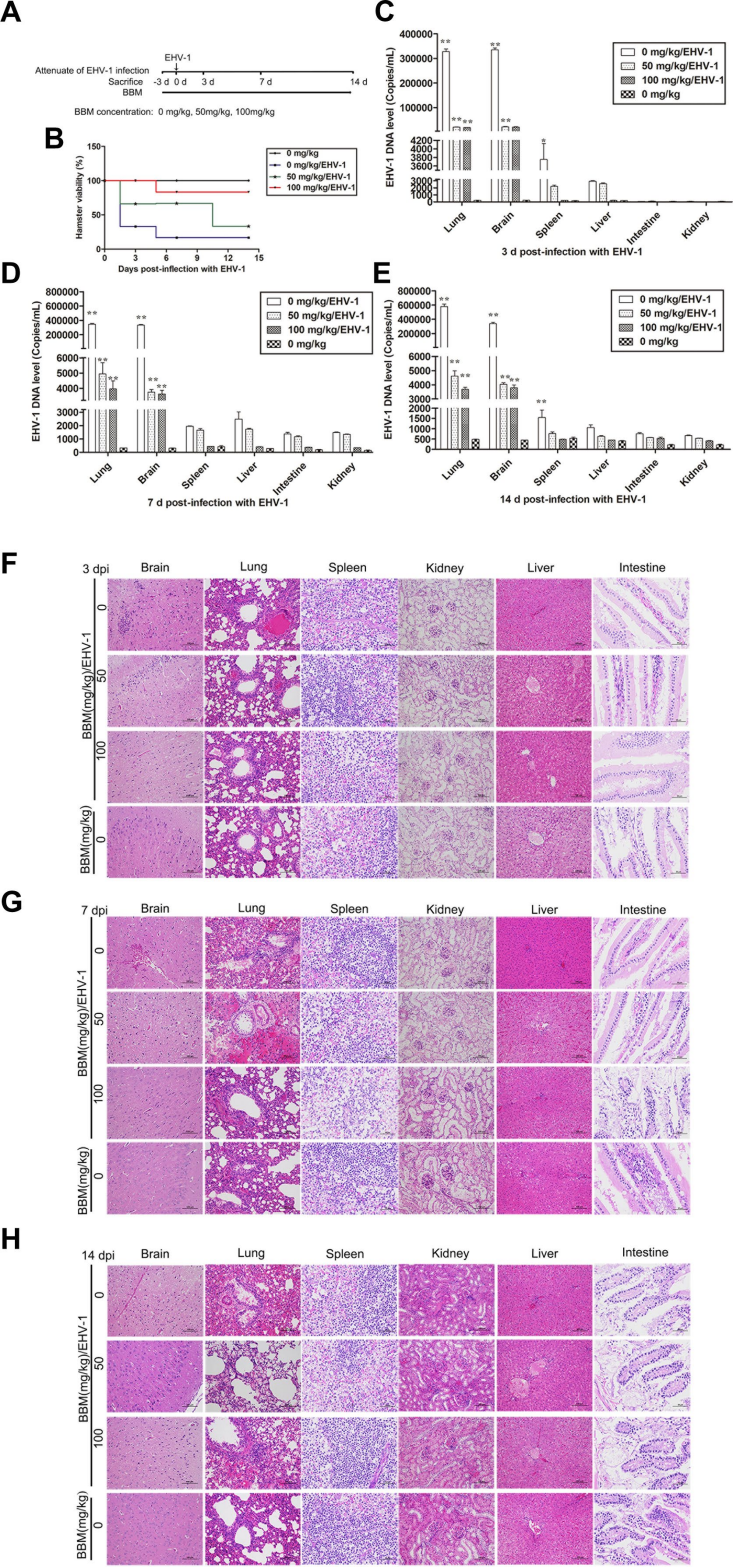
Attenuating effects of BBM on EHV-1 infection. **(A)** Schematic representation of the hamster treatment with BBM and EHV-1. **(B)** Hamster viability after infection with EHV-1. **(C–E)** EHV-1 DNA level at 3, 7, and 14 d pi with EHV-1. **(F–H)** Histopathological manifestations after EHV-1 infection for 3, 7, and 14 d. *P*-values less than 0.05 were considered as statistically significant. *p < 0.05 means significant difference, **p < 0.01 means very significant.

Following pre-treatment with different concentrations of BBM 3 d pi and infection with EHV-1 YM2019 (Figure 4A), the hamsters began to show ruffled fur, dyspnea, crouching and a hunched posture in corners at 2 d pi with EHV-1. The hamsters also exhibited increased reactivity to external stimulation, and sialorrhea. On day 2 pi with EHV-1, the animals showed decreased survival (Figure 4B) and a significant increase in the level of EHV-1 DNA (*p* < 0.01). However, these changes could be reduced by pre-treatment of the hamsters with 50 mg/kg BBM (*p* < 0.05) and significantly reduced by pre-treatment with 100 mg/kg BBM (*p* < 0.01). Furthermore, the changes were dose- and time-dependent, especially in brain and lung tissues, and there was little difference in the other tissues (Figures 4C–E). EHV-1 replication predominantly induced changes in the brain and lungs (Table 1). Analysis of the pathological tissue sections showed that EHV-1 infection led to infiltration of neutrophils and plasma cells in the meninges (meningitis), severe lymphocytic meningoencephalitis, the OB were expanded by macrophages and lymphocytes, the alveolar septa exhibited diffuse thickening with macrophages and a few neutrophils and lymphocytes, and there was multifocally amorphous eosinophilic fluid (edema) with erythrocytes (hemorrhage) in the alveolar lumen. The 50 mg/kg BBM group experienced a reduction in these histopathological characteristics; occasionally, there was an increase in erythrocytes in the alveolar lumen with contained eosinophilic, Multifocally, within there was degeneration, necrosis cells to the lung. The hamsters that received 100 mg/kg BBM had no histopathological symptoms (Figures 4F–H). Other tissues did not exhibit any significant pathological changes expect a little hemorrhage.

**Table 1:** Lesion of histopathological manifestations in BBM-pretreated hamster infected with EHV-1 at different intervals.

Concentration (mg/kg)	Brain	Lung	Liver	Spleen	Kidney	Intestine	Brain	Lung	Liver	Spleen	Kidney	Intestine
	3 d						7 d					
0	+	+	−	+	−	−	++	+	−	−	−	−
50	+	+	−	−	−	−	+	+	−	−	−	−
100	−	−	−	−	−	−	−	−	−	−	−	−
	14 d											
0	++	+++	+	+	−	−						
50	++	++	−	−	−	−						
100	+	−	−	−	−	−						

“−” no lesion; “+” mild; “++” moderate; “+++” severe.

### 3.5. BBM reduced EHV-1 replication *in vivo*

**Figure 5:**
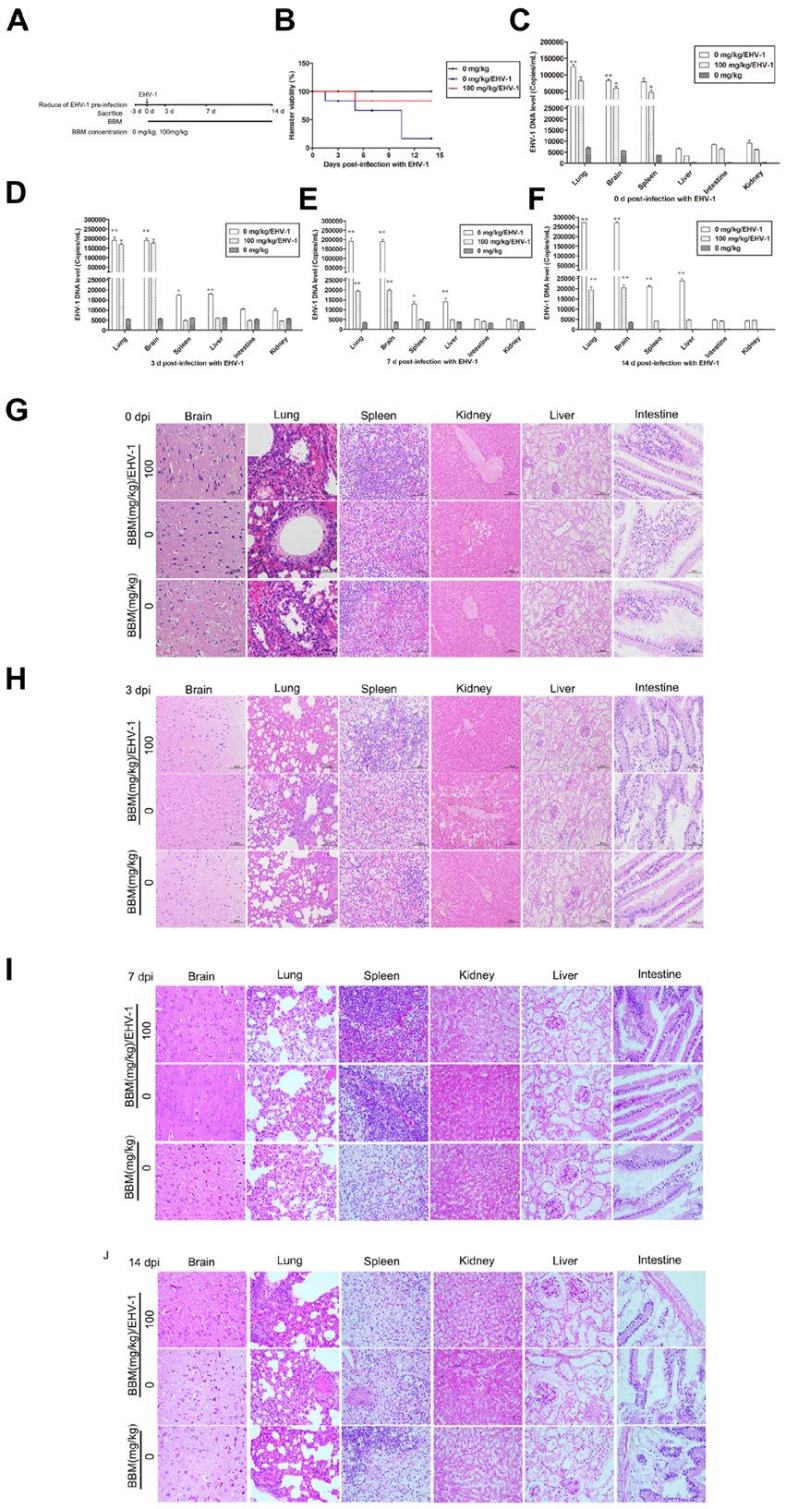
Effects of BBM on EHV-1 replication *in vivo*. **(A)** Schematic representation of the hamster treatment with BBM and EHV-1. **(B)** Hamster viability after infection with EHV-1. **(C-F)** EHV-1 DNA level at 0, 3, 7, and 14 d pi with EHV-1. **(G-J)** Histopathological manifestations after EHV-1 infection at 0, 3, 7, and 14 d pi. *P*-values less than 0.05 were considered as statistically significant. *p < 0.05 means significant difference, **p < 0.01 means very significant.

Hamsters were infected with EHV-1 YM2019 for 3 d and then treated with different concentrations of BBM (Figure 5A). The hamsters began to show severe neurological symptoms with difficulty breathing and increased reactivity to external stimulation. In addition, the animals showed decreased survival after EHV-1 infection (Figure 5B). The level of EHV-1 DNA was significantly increased in EHV-1-infected hamsters (*p* < 0.01), but this was reduced in hamsters that received 50 mg/kg BBM (*p* < 0.05) and significantly reduced in those that received 100 mg/kg BBM (*p* < 0.01). All reductions in the level of EHV-1 DNA by BBM were time- and dose-dependent, especially in the brain and lung; there was a lower level of EHV-1 DNA in the spleen and liver, but not in the kidneys and intestine (Figures 5C–F). EHV-1 infection leads to marked tissue damage in the brain, lung, spleen, liver, and a little in the intestine, with hardly any tissue damage in the kidneys (Table 2). Histopathological section analysis showed that EHV-1 infection could increase connective tissue in the liver and red pulp in the spleen, the OB were expanded by macrophages and lymphocytes, the alveolar septa exhibited diffuse thickening with macrophages and a few neutrophils and lymphocytes, and there was multifocally amorphous eosinophilic fluid (edema) with erythrocytes (hemorrhage) in the alveolar lumen. Occasionally, erythrocyte increased in the alveolar lumen with contained eosinophilic, Multifocally, within there was degeneration, necrosis cells to the lung, while the hamsters that received 100 mg/kg BBM had no histopathological symptoms (Figures 5G–J). There was no significant pathological changes expect a little hemorrhage in the intestine and kidneys. These results demonstrated that BBM significantly inhibited EHV-1 infection in Syrian hamsters. Inflammatory cells increased in the brain, and neutrophils, giant cells, and erythrocytes increased transference cure. BBM significantly inhibited EHV-1 and histopathological changes, indicating that BBM significantly affected EHV-1 pre-infection in Syrian hamsters.

**Table 2:** Lesion of histopathological manifestations in BBM treated hamster infected with EHV-1 at different intervals.

Concentration (mg/kg)	Brain	Lung	Liver	Spleen	Kidney	Intestine	Brain	Lung	Liver	Spleen	Kidney	Intestine
	0 d						3 d					
0	+++	+++	−	−	−	−	+++	+++	−	−	−	−
100	+++	+++	−	−	−	−	++	+	−	−	−	−
	7 d						14 d					
0	+++	+++	−	−	−	−	+++	+++	+	+	−	−
100	+	+	−	−	−	−	−	−	−	−	−	−

“−” o lesion; “+” mild; “++” moderate；“+++” severe.

## 4. Discussion

This work assessed the potential of the antiviral activity of BBM to inhibit EHV-1 infection *in vitro* and *in vivo* (hamsters). BBM is a natural, potent, pharmacologically active biomolecule isolated from *Berberis amurensis*. BBM can modulate oncogenic cell signaling pathways in different cancers, and BBM hydrochloride is currently being used clinically as an anti-inflammatory agent. However, in recent years, the use of BBM as an antiviral drug has been reported. A study by Lihong Huang et al. (27) found that BBM inhibits SARS-CoV-2 infection by compromising TRPML-mediated endolysosomal trafficking of ACE2, and a study by Yi (14) found that repurposing BBM hydrochloride to inhibit Ebola virus by targeting viral glycoprotein offered a theoretical basis for exploiting new broad-spectrum antiviral drugs. Tong (28) conducted a high-content chemical screening using an FDA-approved drug library and discovered that BBM hydrochloride showed potential antiviral activities against SARS-CoV-2. There is evidence in the literature showing that SARS-CoV-2 infection induces calcium release from the infected cells, and the compound nicosamide, which inhibits the calcium-activated ion channel TMEM16F, can efficiently block SARS-CoV-2 S-induced syncytia and virus infection (29). Therefore, as a calcium channel inhibitor, BBM hydrochloride may have a similar antiviral mechanism against SARS-CoV-2 by blunting the calcium oscillations caused by virus infection. To date, the mechanisms of BBM antiviral activity have been studied only in RNA viruses. Jun Wang showed that BBM inhibited bovine viral diarrhea virus (BVDV) infection by inhibiting BVDV-induced autophagy in cells, and its inhibitory effect was obvious in the viral attachment and release stages (30). This was the same as our results that the antiviral ability of BBM inhibited EHV-1 infection in cells by impeding attachment. Furthermore, we found that BBM inhibits EHV-1 infection by disrupting internalization and reducing tissue damage caused by EHV-1 infection in hamsters. We will verify these speculations in future work to elucidate the antiviral mechanism of BBM hydrochloride against EHV-1.

In this study, the SYBR Green qPCR method was used to verify that BBM inhibits EHV-1 replication. This method not only avoids sample errors caused by contamination but also has better sensitivity and accuracy compared with traditional methods for measuring viral replication. Samples treated with 10 μM of BBM exhibited significant inhibition of EHV-1 replication after 24 h, as measured by qPCR, with the greatest inhibitive effect of approximately 50 times (compared with untreated samples) at 36 h, which may be dependent on the living curve. EHV-1 YM2019 has a rapid growth period and logarithmic replication from 12 h to 48 h. In addition, the largest toxicity titer occurs at 36 h and 48 h; thus, the most obvious inhibitive effect of BBM occurred during this period. However, this effect has not been confirmed in other DNA viruses.

Recently, several animal models for EHV-1 infection have been developed, many of which use mice and Syrian hamsters (31, 32). In our study, hamsters were euthanized, and tissues were used in viral DNA detection. BBM significantly inhibited EHV-1 replication in some tissues, such as the brain and lungs, and this was congruent with the findings of Kasem (33), and the pathological tissue tended to normal. These changes were dependent on the dose and time of BBM administration. To verify that BBM inhibited EHV-1 replication, tissues treated with different concentrations of BBM (0, 50, or 100 mg/kg) were collected and analyzed, and the results showed that BBM significantly inhibited EHV-1 replication from 3 d to 14 d, as measured by qPCR. Overall, we demonstrated that BBM treatment significantly inhibited EHV-1 replication. This provides a new basis for the attenuation and reduction of pre-infection of EHV-1. Sometimes phenomena regarding the efficacy of antiviral drugs *in vivo* have been observed that are not consistent with *in vitro* findings; consequently, the effects of BBM need to be further studied in horses.

## 5. Conclusion

This study showed a favorable effect of BBM in controlling EHV-1 infection *in vitro* and in hamsters, and these results support our hypothesis that BBM can be used as an effective drug to inhibit the replication of EHV-1 YM2019, which is a DNA virus.

## Data availability statement

The original contributions presented in the study are included in the article/supplementary material, further inquiries can be directed to the corresponding authors.

## Ethics statement

The animal study was reviewed and approved by Animal Welfare and Ethics Committee of Xinjiang Agricultural University.

## Author contributions

QF, ZL, and HS contributed to conception and design of the study. YHe organized the database. ZL performed the statistical analysis. ZL wrote the first draft of the manuscript. LG, RQ, RS, YHu, JL, DR, and JC wrote sections of the manuscript. QF and HS participate in article writing, as final approval of the version to be published, and agreement to be accountable for all aspects of the work in ensuring that questions related to the accuracy or integrity of any part of the work are appropriately investigated and resolved. All authors contributed to the article and approved the submitted version.

## Funding

This work was supported by Natural Science Foundation of Xinjiang Uygur Autonomous Region (grant no. 2022D01E15), Xinjiang Uygur Autonomous Region Youth Science and Technology Top Talents Project (grant no. 2022TSYCCX0049), International Science and Technology Cooperation Program of Xinjiang Uygur Autonomous Region (grant no. 2020E01006).

## Conflict of interest

The authors declare that the research was conducted in the absence of any commercial or financial relationships.

## Publisher’s note

All claims expressed in this article are solely those of the authors and do not necessarily represent those of their affiliated organizations, or those of the publisher, the editors and the reviewers. Any product that may be evaluated in this article, or claim that may be made by its manufacturer, is not guaranteed or endorsed by the publisher.

## References

[1] Walter J Balzer HJ Seeh C Fey K Bleul U Osterrieder N. Venereal shedding of equid herpesvirus-1 (EHV-1) in naturally infected stallions. J Vet Intern Med. (2012) 26:1500–4. doi: 10.1111/j.1939-1676.2012.00997.x 22947047

[2] Sutton G Garvey M Cullinane A Marcillaud PC. Molecular surveillance of EHV-1 strains circulating in France during and after the major 2009 outbreak in Normandy involving respiratory infection, neurological disorder, and abortion. Viruses. (2019) 11:916. doi: 10.3390/v11100916, PMID: 31590336 PMC6832873

[3] Oladunni FS Horohov DW Chambers TM. EHV-1: a constant threat to the horse industry. Front Microbiol. (2019) 10:2668. doi: 10.3389/fmicb.2019.02668, PMID: 31849857 PMC6901505

[4] Stokol T Soboll HG. Current research in equid herpesvirus Type-1 (EHV-1). Front Vet Sci. (2020) 6:492. doi: 10.3389/fvets.2019.00492, PMID: 31998768 PMC6965053

[5] Zarski LM Vaala WE Barnett DC Gisela SH. A live-attenuated equine influenza vaccine stimulates innate immunity in equine respiratory epithelial cell cultures that could provide protection from equine herpesvirus 1. Front Vet Sci. (2021) 8:674850. doi: 10.3892/or_00000688, PMID: 34179166 PMC8224402

[6] Sutton G Normand C Carnet F Couroucé A Garvey M Castagnet S. Equine herpesvirus 1 variant and new marker for epidemiologic surveillance, Europe, 2021. Emerg Infect Dis. (2021) 27:2738–9. doi: 10.3201/eid2710.210704, PMID: 34546162 PMC8462333

[7] Ma G Eschbaumer M Said A Hoffmann B Beer M Osterrieder N. An equine herpesvirus type 1 (EHV-1) expressing VP2 and VP5 of serotype 8 bluetongue virus (BTV-8) induces protection in a murine infection model. PLoS One. (2012) 7:e34425. doi: 10.1371/journal.pone.0034425, PMID: 22511939 PMC3325243

[8] Mc FA Hanlon D McKenzie RK Mayhew I. The first reported outbreak of equine herpesvirus myeloencephalopathy in New Zealand. N Z Vet J. (2016) 64:125–34. doi: 10.1080/00480169.2015.1096853, PMID: 26414406

[9] Si YL Li HL Teh BS Thong Y. Anti-inflammatory and immunosuppressive properties of the bis-benzylisoquinolines: in vitro comparisons of tetrandrine and berbamine. Int J Immunopharmacol. (1989) 11:395–401. doi: 10.1016/0192-0561(89)90086-6, PMID: 2777433

[10] Liu L Liang D Zheng Q Zhao M Lv R Tang J. Berbamine dihydrochloride suppresses the progression of colorectal cancer via RTKs/Akt axis. J Ethnopharmacol. (2023) 303:116025. doi: 10.1016/j.jep.2022.116025, PMID: 36496042

[11] Zhao Y Tan Y Wu G He H. Berbamine overcomes imatinib-induced neutropenia and permits cytogenetic responses in Chinese patients with chronic-phase chronic myeloid leukemia. Int J Hematol. (2011) 94:156–62. doi: 10.1007/s12185-011-0887-7, PMID: 21728004

[12] Liu L Yan J Cao Y Shusheng W. Proliferation, migration and invasion of triple negative breast cancer cells are suppressed by berbamine via the PI3K/Akt/MDM2/p53 and PI3K/Akt/mTOR signaling pathways. Oncol Lett. (2021) 21:70–1. doi: 10.3892/ol.2020.12331, PMID: 33365081 PMC7716707

[13] Huang L Li H Ye Z Yue J. Berbamine inhibits Japanese encephalitis virus (JEV) infection by compromising TPRMLs-mediated endolysosomal trafficking of low-density lipoprotein receptor (LDLR). Emerg Microbes Infect. (2021) 10:1257–71. doi: 10.1080/22221751.2021.1941276, PMID: 34102949 PMC8238074

[14] Cen S Yi D Li Q Shan C. Repurposing of berbamine hydrochloride to inhibit Ebola virus by targeting viral glycoprotein. Acta Pharm Sin B. (2022) 12:4378–89. doi: 10.1016/j.apsb.2022.05.023, PMID: 36561997 PMC9764067

[15] Mesquita LP Arévalo AF Zanatto DA Miyashiro SI Cunha EMS Souza MDCC. Equine herpesvirus type 1 induces both neurological and respiratory disease in Syrian hamsters. Vet Microbiol. (2017) 203:117–24. doi: 10.1016/j.vetmic.2017.03.007, PMID: 28619133

[16] Saleh AG El HN Abd HA Abas OM Anwar S Fukushi H. Comparative study of the pathogenesis of Rhinopneumonitis induced by intranasal inoculation of hamsters with equine Herpesvirus-9, equine Herpesvirus-1 strain Ab4p and Zebra-borne equine Herpesvirus-1. J Comp Pathol. (2020) 180:35–45. doi: 10.1016/j.jcpa.2020.08.002, PMID: 33222872

[17] Zhang D Bai C Ge K Yong W. Establishment of an SYBR green-based real-time PCR assay for porcine circovirus type 4 detection. J Virol Methods. (2020) 285:113963. doi: 10.1016/j.jviromet.2020.113963, PMID: 32882322

[18] Zhu J Huang L Gao F Jian W Chen H Liao M. Berbamine hydrochloride inhibits African swine fever virus infection *in vitro*. Molecules. (2022) 28:170. doi: 10.3390/molecules28010170 36615369 PMC9822360

[19] Grela E Kozłowska J Grabowiecka A. Current methodology of MTT assay in bacteria–a review. Acta Histochem. (2018) 120:303–11. doi: 10.1016/j.acthis.2018.03.007, PMID: 29606555

[20] Álvarez D Muñoz AL Tavera RP Marcela MR. Low neutralizing antibody titers against the mu variant of SARS-CoV-2 in 31 BNT162b2 vaccinated individuals in Colombia. Vaccine. (2022) 10:180. doi: 10.3390/vaccines10020180, PMID: 35214639 PMC8876570

[21] Ran D L Hu Y Liu J H (2020). Application of Equine Herpesvirus Type 1. Xin Jiang: CN110885794A, 2020-03-17. Available at: https://kns.cnki.net/kcms2/article/abstract?v=kxaUMs6x7-4I2jr5WTdXti3zQ9F92xu0ManZHCyoNk-lwS3y-OLIR-85EP8qkznb_EhVOXUVpbQauMwiCIHMky1Fo80uZqFX&uniplatform=NZKPT

[22] Beurden SJ Voorbergen MA Roozenburg I Boerlage AS Haenen OL Engelsma MY. Development and validation of a two-step real-time RT-PCR for the detection of eel virus European X in European eel, *Anguilla anguilla*. J Virol Methods. (2011) 171:352–9. doi: 10.1016/j.jviromet.2010.11.019, PMID: 21126538

[23] Hu Y Jia Q Liu J Ran D. Molecular characteristics and pathogenicity of an equid alphaherpesvirus 1 strain isolated in China. Virus Genes. (2022) 58:284–93. doi: 10.1007/s11262-022-01910-y, PMID: 35567668

[24] Chan CM Chu H Zhang AJ Kwok YY. Hemagglutinin of influenza a virus binds specifically to cell surface nucleolin and plays a role in virus internalization. Virology. (2016) 494:78–88. doi: 10.1016/j.virol.2016.04.008, PMID: 27085069

[25] Lee K Han DG Kim S Choi E Choi KS. Experimental infection of mice with noncytopathic bovine viral diarrhea virus 2 increases the number of megakaryocytes in bone marrow. Virol J. (2018) 15:115–9. doi: 10.1186/s12985-018-1030-7, PMID: 30055639 PMC6064063

[26] Seong G Oem JK Lee KH Choi KS. Experimental infection of mice with bovine viral diarrhea virus. Arch Virol. (2015) 160:1565–71. doi: 10.1007/s00705-015-2412-4, PMID: 25850760

[27] Huang L Yuen TTT Ye Z Yue J. Berbamine inhibits SARS-CoV-2 infection by compromising TRPMLs-mediated endolysosomal trafficking of ACE2. Signal Transduct Target Ther. (2021) 6:168–3. doi: 10.1038/s41392-021-00584-6, PMID: 33895782 PMC8065329

[28] Tong Q Liu G Sang X Zhu X Fu X Dou C. Targeting RNA G-quadruplex with repurposed drugs blocks SARS-CoV-2 entry. PLoS Pathog. (2023) 19:e1011131. doi: 10.1371/journal.ppat.1011131, PMID: 36701392 PMC9904497

[29] Braga L Ali H Secco I Chiavacci E Neves G Goldhill D. Drugs that inhibit TMEM16 proteins block SARS-CoV-2 spike-induced syncytia. Nature. (2021) 594:88–93. doi: 10.1038/s41586-021-03491-6, PMID: 33827113 PMC7611055

[30] Wang J Yang G Zhang L Zhang J Wang J Zou Y. Berbamine hydrochloride inhibits bovine viral diarrhea virus replication via interfering in late-stage autophagy. Virus Res. (2022) 321:198905. doi: 10.1016/j.virusres.2022.198905, PMID: 36064041

[31] Stokes A Allen GP Pullen LA Murray PK. A hamster model of equine herpesvirus type 1 (EHV-1) infection; passive protection by monoclonal antibodies to EHV-1 glycoproteins 13, 14 and 17/18. J Gen Virol. (1989) 70:1173–83. doi: 10.1099/0022-1317-70-5-1173, PMID: 2471805

[32] Awan AR Chong YC Field HJ. The pathogenesis of equine herpesvirus type 1 in the mouse: a new model for studying host responses to the infection. J Gen Virol. (1990) 71:1131–40. doi: 10.1099/0022-1317-71-5-1131 2161048

[33] Kasem S Yu MHH Yamada S Hideto F. The ORF37 (UL24) is a neuropathogenicity determinant of equine herpesvirus 1 (EHV-1) in the mouse encephalitis model. Virology. (2010) 400:259–70. doi: 10.1016/j.virol.2010.02.012, PMID: 20199788

